# Dietary Intake of Vitamin B_6_ and Risk of Breast Cancer in Taiwanese Women

**DOI:** 10.2188/jea.JE20100188

**Published:** 2011-09-05

**Authors:** Yu-Ching Chou, Chi-Hong Chu, Mei-Hsuan Wu, Giu-Cheng Hsu, Tsan Yang, Wan-Yun Chou, Hsin-Ping Huang, Meei-Shyuan Lee, Cheng-Ping Yu, Jyh-Cherng Yu, Chien-An Sun

**Affiliations:** 1 School of Public Health, National Defense Medical Center, Taipei, Taiwan; 2 Department of Surgery, Tri-Service General Hospital, Taipei, Taiwan; 3 Department of Public Health, College of Medicine, Fu-Jen Catholic University, Taipei, Taiwan; 4 Department of Radiology, Tri-Service General Hospital, Taipei, Taiwan; 5 Department of Health Business Administration, Meiho University, Pingtung, Taiwan; 6 Department of Pathology, Tri-Service General Hospital, Taipei, Taiwan

**Keywords:** breast cancer, ER-defined breast tumors, 1-carbon metabolism, vitamin B_6_

## Abstract

**Background:**

B vitamins, including vitamin B_6_, are coenzymes that are important for DNA integrity and stability. Deficiencies in B vitamins may promote tumor carcinogenesis.

**Methods:**

We examined the association of dietary vitamin B_6_ intake with overall breast cancer risk and breast cancers stratified by hormone receptor status. This case-control study included 391 breast cancer cases and 782 control subjects enrolled at the Tri-Service General Hospital in Taipei, Taiwan. Energy-adjusted intake of vitamin B_6_ was derived from a food frequency questionnaire. Odds ratios (ORs) and 95% confidence intervals (CIs) were estimated using logistic regression.

**Results:**

As compared with women in the lowest tertile, the multivariate-adjusted ORs for breast cancer among women in the second and highest tertiles of vitamin B_6_ intake were 0.78 (95% CI, 0.64–2.52) and 0.64 (0.26–0.92), respectively. In addition, higher vitamin B_6_ intake was associated with a significantly lower risk of developing ER-negative breast tumors.

**Conclusions:**

Our findings suggest that higher intake of vitamin B_6_ is associated with a reduction in breast cancer risk, particularly ER-negative tumors.

## INTRODUCTION

Breast cancer is the most commonly diagnosed cancer in Taiwanese women, and age at tumor onset is younger in this population.^1^ Recent efforts have focused on the role of dietary factors in the etiology of hormone-dependent breast carcinogenesis. However, epidemiologic studies of the relationship of dietary factors with breast cancer risk in Taiwanese women are relatively rare. B vitamins such as folate, vitamin B_6_, and vitamin B_12_ play important roles as coenzymes in 1-carbon metabolism, which is critical for nucleotide synthesis and DNA methylation.^2^ Aberrations in nucleotide synthesis or DNA methylation can contribute to carcinogenesis.^3^ Among the nutrients involved in 1-carbon metabolism, vitamin B_6_ is a crucial coenzyme in the conversion of tetrahydrofolate to 5,10-methylenetetrahydrofolate,^2^ which is needed for nucleotide synthesis in DNA synthesis, repair, and methylation. Thus, inadequate vitamin B_6_ intake might lead to imbalances in DNA precursors, disruption in DNA repair, and aberrations in DNA methylation, any of which might enhance carcinogenesis. Apart from its role in the synthesis, repair, and methylation of DNA, vitamin B_6_ is necessary for the synthesis of glutathione from homocysteine via cystathionine and cysteine.^4^ Glutathione is a cofactor of the glutathione *S*-transferases and glutathione peroxidase, which function in the detoxification of many carcinogenic compounds and in the protection of cells from oxidative DNA damage.^5^ Vitamin B_6_ has been shown to reduce oxidative stress as well as cell proliferation and angiogenesis, and moderate doses of vitamin B_6_ have been shown to suppress colorectal carcinogenesis in mice given injections of a carcinogen.^4^^,^^6^

Despite its importance in 1-carbon metabolism and the reduction of oxidative stress, there is limited epidemiologic evidence of an association of vitamin B_6_ intake or circulating concentrations of pyridoxal 5′-phosphate (PLP, the principal active form of vitamin B_6_) with the risk of carcinogenesis, especially in the breast. To gain a better understanding of the role of vitamin B_6_ in breast cancer risk, we conducted a case-control study of the association between dietary intake of vitamin B_6_ and breast cancer risk in Taiwanese women. In addition, Zhu and Williams hypothesized^7^ that estrogen receptor (ER)-negative and perhaps progesterone receptor (PR)-negative breast tumors, which result from hypermethylation of the promoter region of ER and PR, respectively,^8^ are linked to low intake of B vitamins. Thus, we also examined the association with respect to the hormone receptor status of breast tumors.

## METHODS

### Identification of case and control subjects

This study is an extension of a previously reported case-control study.^9^^–^^13^ The study comprised subjects attending the Department of Surgery and health examination clinics at the Tri-Service General Hospital Taipei, Taiwan from January 2004 to June 2008. Using hospital chart numbers, 391 women aged 24 to 72 years (the cases) were consecutively selected from subjects with a first confirmed histopathologic diagnosis of breast carcinoma. These patients accounted for 91.7% of women with breast cancer who attended our breast cancer clinics during the study period. More importantly, because the clinics in which this study was performed are major breast cancer clinics in northern Taiwan, our patients accounted for a sizable proportion (approximately 20%) of all breast cancer cases diagnosed during the study period in this region.^14^ The histopathologic profile included 282 cases of invasive ductal carcinoma, 42 cases of invasive lobular carcinoma, and 67 cases of carcinoma in situ. There were 227 premenopausal and 164 postmenopausal breast cancer cases. Data on breast tumor ER and PR status were obtained primarily from the hospital laboratory that routinely conducts immunohistochemical assays to determine steroid receptor status of tumor tissues from breast cancer patients. Immunohistochemical assays were interpreted as positive (presence of antibody nuclear staining) or negative by pathologists who recorded this result directly on the pathology report. The ER and PR status were obtained for 95% (371 of 391) of the breast cancer cases: 268 of the cases were ER-positive and 295 were PR-positive. The control subjects were women with no history of cancer and were simultaneously recruited from the health examination clinics of the same hospital during the same study period. They underwent a 1-day comprehensive health examination (including regular breast cancer screening using X-ray mammography and ultrasonic examination), and those with any evidence of breast cancer, suspicious precancerous lesions of the breast, or other cancers were excluded from the control group. Because the examination was not sponsored by the National Insurance Program, the controls might represent a group of women who have greater concern for their health. Approximately 80.4% of women who were initially identified as potential controls participated in the study, and these participants accounted for approximately 50% of all women attending the clinic. No significant differences in breast cancer risk factors were found between the included and excluded control subjects. Two control subjects were matched to each case by date of enrollment (±3 months) and duration of fasting (±4 hours). Overall, 391 breast cancer cases and 782 matched controls were included in the analysis. This study was conducted according to the guidelines specified in the Declaration of Helsinki, and all procedures involving human subjects were approved by the Institutional Review Board of the Tri-Service General Hospital in Taipei (approval number: TSGHIRB 096-05-158). Written informed consent was obtained from all subjects.

### Collection of questionnaire data

All participants underwent a personal interview administered by well-trained interviewers in conformity with institutional guidelines for studies including human subjects. Trained interviewers collected information on sociodemographic characteristics, menstrual and reproductive history, menopausal status, lifestyle behaviors, and medical history. In this study, postmenopausal was defined as absence of menses during the last 12 months or history of oophorectomy or hysterectomy; no attempt was made to distinguish between artificial and natural menopause. In addition, because the average quantities of cigarettes and alcohol consumed by Chinese women are not large in Taiwan, habitual cigarette smoking was defined as smoking cigarettes at least once a week for more than 1 year. Similarly, habitual alcohol drinking was defined as consuming any alcoholic beverage at least once a week for more than 1 year. A 31-item semi-quantitative food frequency questionnaire (FFQ) was administered to estimate dietary intake among participants. The FFQ was designed to assess usual dietary intake over a 1-year period, ignoring any recent changes. This FFQ inquired about how often participants consumed individual food items (frequency of consumption) and about representative sizes (as compared with standard portions) during either the preceding year (control subjects) or 1 year before their breast cancer diagnosis (cancer patients). Photographs of foods, showing different portion sizes, were used to facilitate quantification of intakes. Nutrient values in foods were computed by multiplying the frequency of responses by the nutrient content of specified portion sizes according to a previously established Taiwanese nutrient database.^15^^–^^17^ The regression-residual model was used to adjust intake of vitamin B_6_, vitamin B_12_, and folate.^18^ Although the FFQ might not be applicable for estimating actual intake of B vitamins among subjects, it is likely to be adequate for ranking individuals by dietary intake of B vitamins. The reliability and validity of the FFQ have been evaluated previously among participants of the MJ Health Screening Center, a population that is comparable to our present study subjects attending health examination clinics.^19^ The intra-class correlation coefficients for vitamin B_6_, vitamin B_12_, and folate intake from FFQ repeated measurements over the 1-year period were 0.644, 0.743, and 0.513, respectively. In addition, the Spearman correlation coefficients between energy-adjusted nutrient intakes estimated from the FFQ and a 4-day dietary record in a subsample of 59 individuals were 0.38 for vitamin B_6_, 0.33 for vitamin B_12_, and 0.30 for folate. Moreover, the validity of the FFQ was further assessed in the present study on the basis of laboratory analyses of biochemical indicators of dietary intake^20^ in a randomly selected subsample of the control group (*n* = 89). The Spearman correlation coefficient between vitamin B_6_ intake from the FFQ and plasma level of PLP was 0.31. The correlation between folate intake from the FFQ and plasma folate levels was 0.34. These results indicate that the predictive power of our dietary assessment methodology was relatively good. Information on multivitamin supplement use was also requested on the FFQ, but data on nutrient intake from supplements were not included in the analyses. Participants were queried as to whether they had ever taken multivitamin supplements in the preceding year (control subjects) or the 1 year before breast cancer diagnosis (cancer cases). However, information regarding the specific brand, composition, dosage, and duration of use of multivitamin supplements was not available. Accordingly, use of multivitamin supplements among participants was categorized as never or ever use in the statistical analyses. To avoid the influence of treatment on measurements, questionnaire data were obtained before cases were scheduled for surgery and radio- or chemotherapy.

### Statistical methods

Differences between cases and controls in the distributions of age at menarche, age at first full-term pregnancy (FFTP), age at menopause, parity, cigarette smoking, alcohol drinking, use of multivitamin supplements, menopausal status, postmenopausal hormone use, and family history of breast cancer were evaluated using the chi-square test. For statistical analysis of data on B vitamin intake, including vitamin B_6_, vitamin B_12_, and folate, we used tertile cut points to categorize B vitamins intake into 3 groups, with cut points based on the distributions in the control subjects. Individuals in the lowest tertile were treated as the referent in the statistical analyses. To maintain matched triplet integrity, we used conditional logistic regression to estimate odds ratios (ORs) and 95% confidence intervals (CIs) for the association between dietary intake of B vitamins and breast cancer risk. Age at enrollment (treated as a continuous variable), reproductive risk factors (including age at menarche, age at FFTP, parity, and age at menopause; treated as continuous variables), postmenopausal hormone use, and family history of breast cancer (treated as categorical variables), which had been identified in the literature as potential confounders, were included in the logistic regression model. In addition, to increase statistical power, unconditional logistic regression was used to further examine the association of vitamin B_6_ with breast cancer by level of folate intake, use of multivitamin supplements, menopausal status, and hormone receptor status. Tests for trend were conducted by entering the categoric variables as continuous variables in the model and calculating a Wald statistic. All statistical analyses were performed using SAS statistical software (version 9.1; SAS Institute, Cary, NC, USA). A *P* value less than 0.05 was considered statistically significant.

## RESULTS

The baseline characteristics of patients with breast cancer and control subjects are summarized in Table 1. Cases and controls were similar with regard to age at enrollment, age at menarche, age at FFTP, parity, menopausal status, age at menopause, postmenopausal hormone use, family history of breast cancer, smoking status, and alcohol intake. However, patients with breast cancer were significantly more likely to have postmenopausal hormone therapy and less likely to be users of multivitamin supplements than their matched control subjects.

**Table 1.** Characteristics of breast cancer cases and matched controls

Characteristics	Cases (*n* = 391) No. (%)	Controls (*n* = 782) No. (%)	*P*
Age at enrollment (years)			
<45	113 (28.9)	261 (33.4)	0.30
45–52	140 (35.8)	261 (33.4)	
≥52	138 (35.3)	260 (33.2)	
Age at menarche (years)			
<13	195 (50.4)	377 (48.6)	0.75
13–14	95 (24.5)	206 (26.5)	
≥14	97 (25.1)	193 (24.9)	
Age at FFTP^a^ (years)			
<24	130 (37.1)	282 (40.0)	0.09
24–27	98 (28.0)	223 (31.6)	
≥27	122 (34.9)	200 (28.4)	
Menopausal status			
Premenopausal	227 (58.1)	423 (54.1)	0.20
Postmenopausal	164 (41.9)	359 (45.9)	
Postmenopausal hormone use			
Never	68 (41.5)	200 (55.7)	<0.01
Ever	96 (58.5)	159 (44.3)	
Age at menopause (years)			
<50	75 (46.0)	179 (50.4)	0.40
≥50	88 (54.0)	176 (49.6)	

^a^FFTP, first full-term pregnancy.

Overall, there was no statistically significant difference between cases and controls in terms of total caloric intake (1398.8 ± 378.4 vs 1422.8 ± 410.2 kcal/day, *P* = 0.33). For all women, greater intakes of vitamin B_6_, vitamin B_12_, and folate were associated with lower risk of breast cancer (Table 2). After adjusting for matching variables and potential risk factors of breast cancer, the ORs (95% CIs) for the highest tertile of intake as compared with the lowest were 0.64 (0.26–0.92) for vitamin B_6_, 0.83 (0.73–2.54) for vitamin B_12_, and 0.71 (0.24–2.15) for folate. However, a significant inverse trend was observed only between vitamin B_6_ intake and breast cancer risk (*P* for trend test = 0.04). When breast cancer cases were stratified by diagnosis (in situ vs invasive disease), the inverse trend of ORs for breast cancer in relation to the intake of B vitamins did not differ substantially from that for all women combined (data not shown).

**Table 2.** Breast cancer risk in relation to energy-adjusted dietary intake of B vitamins

	Tertile of energy-adjusted dietary intake of B vitamins^d^		
	
	T1	T2	T3
Vitamin B_6_ (µg/d)	≤580.45	580.46–701.20	>701.20
No. cases/no. ​ controls	159/261	114/260	118/261
OR^a,b,c^ (95% CI^a^)	1.00 (reference)	0.78 (0.64–2.52)	0.64 (0.26–0.92)
*P*_for trend_	0.04		
Vitamin B_12_ (µg/d)	≤5.28	5.29–8.15	>8.15
No. cases/no. ​ controls	126/260	142/262	123/260
OR^a,b,c^ (95% CI^a^)	1.00 (reference)	0.89 (0.53–1.65)	0.83 (0.73–2.54)
*P*_for trend_	0.08		
Folate (µg/d)	≤110.44	110.45–143.42	>143.42
No. cases/no. ​ controls	149/260	124/262	118/260
OR^a,b,c^ (95% CI^a^)	1.00 (reference)	0.46 (0.23–0.92)	0.71 (0.24–2.15)
*P*_for trend_	0.26		

^a^OR, odds ratio; CI, confidence interval.

^b^In addition to the matching variables of date of enrollment and fasting status, ORs were further adjusted for age at enrollment, age at menarche, age at first full-term pregnancy, parity, menopausal status, age at menopause, postmenopausal hormone use, family history of breast cancer, use of multivitamin supplements, and total energy intake.

^c^The conditional logistic regression model was used for multivariate analysis.

^d^Cutoff points in tertiles were selected from the controls.

In subgroup analyses according to menopausal status, the apparent inverse association between vitamin B_6_ intake and breast cancer risk was primarily observed in premenopausal women, and this protective effect seemed to be stronger than that for all women combined. In premenopausal women, the adjusted ORs for the second and highest tertiles of vitamin B_6_ intake as compared with the lowest tertile were 0.53 (95% CI, 0.32–0.79) and 0.50 (0.31–0.88), respectively. In contrast, intake of vitamin B_6_ was not appreciably associated with breast cancer among postmenopausal women (Table 3).

**Table 3.** Risk of breast cancer by tertiles of energy-adjusted dietary vitamin B_6_ intake, stratified by menopausal status

	Tertile of energy-adjusted dietary vitamin B_6_ intake^e^ (µg/d)		
	
	T1	T2	T3
Premenopausal women	≤594.60	594.61–707.06	>707.06
No. cases/no. ​ controls	105/141	60/141	62/141
OR^a,b,c^ (95% CI^a^)	1.00 (reference)	0.53 (0.32–0.79)	0.50 (0.31–0.88)
*P*_for trend_	0.04		
Postmenopausal women	≤565.70	565.71–691.08	>691.08
No. cases/no. ​ controls	55/119	53/120	56/120
OR^c,d^ (95% CI)	1.00 (reference)	0.87 (0.70–1.94)	1.08 (0.79–2.78)
*P*_for trend_	0.23		

^a^OR, odds ratio; CI, confidence interval.

^b^In addition to the matching variables of date of enrollment and fasting status, ORs were further adjusted for age at enrollment, age at menarche, age at first full-term pregnancy, parity, family history of breast cancer, use of multivitamin supplements, and total energy intake.

^c^Unconditional logistic regression was used for multivariate analysis of subjects stratified by menopausal status.

^d^In addition to the matching variables of date of enrollment and fasting status, ORs were further adjusted for age at enrollment, age at menarche, age at first full-term pregnancy, parity, menopausal status, age at menopause, postmenopausal hormone use, family history of breast cancer, use of multivitamin supplements, and total energy intake.

^e^Cutoff points in tertiles were selected from the controls.

It has been proposed that the effects of vitamin B_6_ are modified by other nutrients involved in 1-carbon metabolism and by the use of vitamin supplements.^21^^,^^22^ Therefore, we evaluated whether the observed association between dietary intake of vitamin B_6_ and breast cancer risk differed by level of folate intake and multivitamin supplement use status. As shown in Table 4, a significant inverse trend in ORs for breast cancer in relation to vitamin B_6_ intake was more prominent among women whose diets were low in folate intake (defined by the level of intake below the median value in the control group) and those who did not take multivitamin supplements.

**Table 4.** Association between energy-adjusted dietary vitamin B_6_ intake and breast cancer risk, stratified by dietary folate intake and use of multivitamin supplements

Characteristics	Tertile of energy-adjusted vitamin B_6_ intake^f^ (µg/d)			*P*_for trend_

	T1	T2	T3	
Dietary folate intake (µg/d)^a,b^				
≤122.83	≤480.06	480.07–659.86	>659.86	
No. cases/no. controls	90/127	65/128	50/127	
OR^c,d^ (95% CI^c^)	1.00 (reference)	0.69 (0.36, 1.06)	0.57 (0.36, 0.95)	0.03
>122.83	≤613.38	613.39–829.13	>829.13	
No. cases/no. controls	68/133	58/134	60/133	
OR^d^ (95% CI)	1.00 (reference)	0.77 (0.58, 1.83)	0.89 (0.46, 1.28)	0.32
Use of multivitamin supplements^b^				
Never	≤560.32	560.33–691.41	>691.41	
No. cases/no. controls	66/89	60/89	43/88	
OR^e^ (95% CI)	1.00 (reference)	0.89 (0.54, 1.28)	0.64 (0.42, 0.98)	0.04
Ever	≤587.66	587.67–700.93	>700.93	
No. cases/no. controls	85/172	60/172	77/172	
OR^e^ (95% CI)	1.00 (reference)	0.81 (0.51, 1.29)	0.90 (0.52, 1.56)	0.67

^a^Cutoff points in median were selected from the controls.

^b^Unconditional logistic regression was used for multivariate analysis.

^c^OR, odds ratio; CI, confidence interval.

^d^In addition to the matching variables of date of enrollment and fasting status, ORs were further adjusted for age at enrollment, age at menarche, age at first full-term pregnancy, parity, age at menopause, postmenopausal hormone use, family history of breast cancer, use of multivitamin supplements, and total energy intake.

^e^In addition to the matching variables of date of enrollment and fasting status, ORs were further adjusted for age at enrollment, age at menarche, age at first full-term pregnancy, parity, age at menopause, postmenopausal hormone use, family history of breast cancer, and total energy intake.

^f^Cutoff points in tertiles were selected from the controls.

It has been hypothesized that ER-negative and perhaps PR-negative breast tumors, which result from hypermethylation of the promoter region of ER and PR,^8^ are linked to low intake of B vitamins.^7^ We thus examined the association between vitamin B_6_ intake and breast cancer risk stratified by the ER and PR status of breast tumors (Table 5). It is notable that higher vitamin B_6_ intake was associated with a significantly lower risk of developing ER-negative breast tumors. Using the lowest tertile intake of vitamin B_6_ as reference, the adjusted ORs for ER-negative breast cancer in relation to the second and highest tertiles of vitamin B_6_ intake were 0.78 (95% CI, 0.43–1.42) and 0.62 (0.30–0.98), respectively. In contrast, there was no appreciable inverse trend in risk of ER-positive tumors in relation to vitamin B_6_ intake. Furthermore, the association between vitamin B_6_ intake and breast cancer risk did not significantly differ according to the PR status of breast tumors.

**Table 5.** Association between energy-adjusted dietary vitamin B_6_ intake and breast cancer risk characterized by hormone receptor status of breast tumors

Characteristics	Tertile of energy-adjusted vitamin B_6_ intake^d^ (µg/d)			*P*_for trend_

	T1	T2	T3	
Estrogen receptor status^a^				
Negative	≤571.59	571.60–699.43	>699.43	
No. cases/no. controls	45/68	33/69	25/69	
OR^b,c^ (95% CI^b^)	1.00 (reference)	0.78 (0.43, 1.42)	0.62 (0.30, 0.98)	0.04
Positive	≤573.33	573.34–699.87	>699.87	
No. cases/no. controls	105/178	76/180	87/178	
OR^c^ (95% CI)	1.00 (reference)	0.82 (0.58, 1.23)	0.86 (0.57, 1.34)	0.59
Progesterone receptor status^a^				
Negative	≤565.00	565.01–697.42	>697.42	
No. cases/no. controls	28/51	24/51	24/50	
OR^c^ (95% CI)	1.00 (reference)	0.80 (0.40, 1.61)	0.85 (0.37, 1.94)	0.68
Positive	≤574.95	574.96–699.87	>699.87	
No. cases/no. controls	120/196	85/197	90/197	
OR^c^ (95% CI)	1.00 (reference)	0.84 (0.57, 1.18)	0.90 (0.53, 1.24)	0.38

^a^Unconditional logistic regression was used for multivariate analysis.

^b^OR, odds ratio; CI, confidence interval.

^c^In addition to the matching variables of date of enrollment and fasting status, ORs were further adjusted for age at enrollment, age at menarche, age at first full-term pregnancy, parity, age at menopause, postmenopausal hormone use, family history of breast cancer, use of multivitamin supplements, and total energy intake.

^d^Cutoff points in tertiles were selected from the controls.

## DISCUSSION

In this case-control study, we observed that dietary intake of vitamin B_6_ was associated with a significantly lower risk of breast cancer among Taiwanese women. This inverse association was particularly evident among premenopausal women, women with low folate intake, and women who had never used vitamin supplements. Moreover, analysis of cases stratified by ER status of breast tumors revealed that the reduction in risk was more pronounced among women with ER-negative breast tumors.

There is a substantial body of data supporting the biological plausibility of a protective effect of vitamin B_6_ on breast cancer risk. The folate metabolism pathway has been reported to be associated with breast carcinogenesis.^9^^,^^23^^–^^28^ Vitamin B_6_ acts as a critical coenzyme in 2 different steps in this pathway: in the synthesis of 5,10-methylenetetrahydrofolate, which is critical for synthesis, repair, and methylation of DNA,^2^^,^^3^ and in catabolism of homocysteine to glutathione, which is involved in the detoxification of many carcinogenic compounds and in the protection of cells from oxidative DNA damage.^4^^,^^5^ Laboratory studies using mice suggest that vitamin B_6_ exerts other anticarcinogenic effects by reducing cell proliferation, nitric oxide production, and angiogenesis.^4^^,^^29^ Despite this laboratory evidence, epidemiologic studies examining the relationship of dietary or plasma levels of vitamin B_6_ with breast cancer risk have been unable to provide unequivocal evidence for a protective effect of vitamin B_6_ in preventing breast cancer development. Several investigators have observed, as we did, an inverse association between dietary or plasma levels of vitamin B_6_ and breast cancer risk,^30^^,^^31^ although other studies have not.^32^^–^^35^ The inconsistent data obtained regarding vitamin B_6_ and breast cancer risk might be at least partly due to differences in vitamin B_6_ intake between populations and by lack of adjustment for factors that potentially influence dietary or plasma levels of vitamin B_6_. In the present study, we found that the inverse association between dietary intake of vitamin B_6_ and breast cancer risk was more prominent among women whose diets were low in folate intake and among those who never used vitamin supplements. Indeed, previous studies have shown that the effects of vitamin B_6_ were modified by the intake of other nutrients involved in the 1-carbon metabolism pathway.^24^^,^^32^ In an earlier report, Zhang et al found an inverse association between dietary intake of vitamin B_6_ and the risk of colorectal cancer among women who did not take vitamin supplements.^21^ Furthermore, results from a randomized trial indicated a beneficial effect on cancer risk in participants treated with vitamin B_6_ alone as compared with those assigned to placebo. In contrast, combined folic acid, vitamin B_6_, and vitamin B_12_ treatment had no significant effect on overall risk of total invasive cancer or breast cancer.^36^ Taken together, these findings indicate the possibility of a threshold effect of vitamin B_6_ on cancer risk. During vitamin B_6_ absorption, cellular uptake and the metabolic pathway itself are saturated systems; thus, micronutrient intake beyond a certain level might have little additional effect.

In this case-control study of Taiwanese women, we observed that the inverse relation of vitamin B_6_ to breast cancer risk was stronger among premenopausal women. This contrasts with previous studies, which noted that the inverse association between vitamin B_6_ and breast cancer risk was primarily present in postmenopausal women.^30^^,^^31^ This apparent contraindication may be explained by the unique characteristics of breast cancer in Taiwanese women, ie, the age of onset in Taiwanese women is much younger than in women from Western countries. Indeed, 29.3% of Taiwanese breast cancer patients develop early-onset (age ≤40 years) breast cancer.^1^^,^^37^ The biological mechanism that explains the effect modification by menopausal status in our data is that cell proliferation in breast epithelial cells is much higher in premenopausal women than in postmenopausal women,^38^ and cells that are dividing and differentiating may be particularly susceptible to aberrations in DNA synthesis, repair, and methylation. Vitamin B_6_ plays a critical role in these processes. Thus, if vitamin B_6_ is important in breast carcinogenesis, it is likely to be more strongly associated with premenopausal breast cancer than with postmenopausal breast cancer. Further studies of larger numbers of both premenopausal and postmenopausal women is needed to help clarify this issue.

Another interesting finding from this study is that higher dietary intake of vitamin B_6_ was associated with significantly lower risk of developing ER-negative breast cancer among women. B vitamins such as folate and vitamin B_6_ play an important role in DNA methylation,^2^^,^^3^ and molecular studies show that ER-negative breast cancer is caused by the lack of ER gene transcription due to the methylation of the CpG island 5′ to the gene.^39^ Likewise, previous epidemiologic studies have shown an elevated risk of ER-negative tumors among women who had a low intake of folate.^23^^,^^24^ Our data and previous study results support the hypothesis that low B vitamin status promotes breast carcinogenesis, at least in part, through its influence on DNA methylation. However, our study found that the association between vitamin B_6_ intake and breast cancer risk did not differ according to PR status, which suggests that the effects of vitamin B_6_ may be more specific to ER.

A notable result of the present investigation is that multivitamin supplement use was common among study subjects. Indeed, commercially available vitamin supplements in Taiwan have seen tremendous growth during the last decade.^40^ Previous studies have reported that 35% to 44% of women in Taiwan regularly consume 1 or more vitamin/mineral supplements.^41^^,^^42^ Our observation suggests that vitamin supplement use may become more common among women in Taiwan. Vitamin supplement use is a marker of a healthy lifestyle in many populations.^43^^,^^44^ In this study, the enrollment of control subjects who were selected from people presenting to a health examination clinic might have overrepresented vitamin supplement use.

Diet represents a complex set of exposures, and the action of a dietary compound is strongly affected by the biochemical milieu in which it resides.^45^^,^^46^ Adding to this complexity is the intercorrelations among numerous foods and nutrients, which result in interactions that are difficult to isolate.^47^ Thus, many dietary components and lifestyle factors may potentially confound the relation of a single dietary component (ie, vitamin B_6_) with breast cancer risk observed in the present study. The results of this study should therefore be interpreted with caution.

Several limitations of this study warrant mention. First, control subjects were selected from attendees of health examination clinics and might represent a group of women who are more concerned about their health. However, the case and control groups were recruited from the same hospital, and the control group comprised individuals who would likely be diagnosed at that hospital were they to develop the disease of interest.^48^ Second, although high participation rates among both eligible case and control subjects minimized potential selection bias, cancer-free women attending health examinations might have dietary habits that differ from those of the general population due to greater health consciousness or disease concerns, which might have led to selection bias and underestimation of the nutrient–cancer risk association. Third, measurement error in a FFQ is an issue for all study designs. Errors in the values reported on FFQs could profoundly affect the results of our case-control study. However, FFQs have been shown to accurately rank the relative intake of micronutrients among individuals and are therefore an adequate, albeit not perfect, instrument for measuring relative dietary intake.^49^^,^^50^ Fourth, dietary intakes assessed in case-control studies are sensitive to recall bias. In particular, as compared with controls, case subjects might have been more motivated to recall their diet, depending on their beliefs regarding the association between diet and their disease. We aimed to limit this bias by recruiting incident cases and interviewing them at an early stage of their disease—and before their acceptance of treatment—thus reducing the likelihood of dietary changes resulting from a cancer diagnosis and subsequent treatment effects. Finally, because our study was based on a small-scale case-control comparison and many subgroup analyses were performed in the study, our findings are subject to chance.

In conclusion, our data indicate that higher dietary intakes of vitamin B_6_ are associated with the risk of developing premenopausal breast cancer and ER-negative breast tumors. These observations suggest a possible complex role for this nutrient in breast cancer development. Further prospective follow-up studies are needed to confirm our results and to elucidate the mechanisms for the observed inverse associations of vitamin B_6_ intake with the risk of developing premenopausal breast cancer and ER-negative breast tumors.
